# Chemistry and Applications of Polysaccharide Solutions in Strong Electrolytes/Dipolar Aprotic Solvents: An Overview

**DOI:** 10.3390/molecules18011270

**Published:** 2013-01-21

**Authors:** Omar A. El Seoud, Haq Nawaz, Elizabeth P. G. Arêas

**Affiliations:** Institute of Chemistry, University of São Paulo, P.O. Box 26077, São Paulo 05513-970, Brazil

**Keywords:** cellulose, chitin, chitosan, starch, cellulose esters, cellulose ethers, homogeneous reaction conditions, regioselectivity of substitution

## Abstract

Biopolymers and their derivatives are being actively investigated as substitutes for petroleum-based polymers. This has generated an intense interest in investigating new solvents, in particular for cellulose, chitin/chitosan, and starch. This overview focuses on recent advances in the dissolution and derivatization of these polysaccharides in solutions of strong electrolytes in dipolar aprotic solvents. A brief description of the molecular structures of these biopolymers is given, with emphases on the properties that are relevant to derivatization, namely crystallinity and accessibility. The mechanism of cellulose dissolution is then discussed, followed by a description of the strategies employed for the synthesis of cellulose derivatives (carboxylic acid esters, and ethers) under homogeneous reaction conditions. The same sequence of presentation has been followed for chitin/chitosan and starch. Future perspectives for this subject are summarized, in particular with regard to compliance with the principles of green chemistry.

## 1. Scope of the Overview

This overview focuses on recent advances in the dissolution and derivatization of cellulose, chitin/chitosan, and starch in solutions of strong electrolytes dissolved in dipolar aprotic solvents, in particular *N,N*-dimethylacetamide (DMAC), and dimethylsulfoxide (DMSO). These solvents induce swelling, but do not dissolve some of the above-mentioned biopolymers, namely cellulose and chitin. Addition of certain strong electrolytes, e.g., LiCl or quaternary ammonium fluoride hydrates, however, leads to the formation of clear polysaccharide solutions. This visual aspect does not necessarily mean the formation of molecularly dissolved biopolymer solutions. Aggregate formation has been experimentally demonstrated and is relevant to the accessibility of the hydroxyl groups of the polysaccharide. Consequently, different experimental conditions are required, e.g., for the derivatization of fibrous celluloses, as compared with those of its microcrystalline (MCC) counterpart. The need for considering the physico-chemical characteristics of the polysaccharide solutions is, therefore, justified. With this background, we present here an overview on the dissolution and derivatization of cellulose, chitin/chitosan, and starch by strong electrolytes in the above-mentioned solvents. To our knowledge, this is the first time that derivatization of these polysaccharides is jointly reviewed. After discussing the relevance of the subject to green chemistry, we consider briefly the structures of the above-mentioned polysaccharides. We discuss the outlines of their derivatization under heterogeneous-(industrial) as well as homogeneous reaction conditions. The part on obtaining polysaccharide solutions includes the strategies employed for biopolymer activation, where required, and the mechanism of dissolution. We then discuss the synthetic strategies and reaction conditions that are usually employed for derivatization, and list the results obtained. We dwell more on cellulose derivatives, in particular the esters and ethers, because their synthesis has received more attention; they are also industrially applied on much wider scale than chitin/chitosan and/or starch.

## 2. Introduction: Relevance to Green Chemistry

The development of polymer technology and consequent increase in world production of petroleum-based polymers has unquestionably resulted in important benefits for diverse industrial sectors. At present, fossil resources, such as petroleum and coal, account for *ca*. 86% of energy and 96% of organic chemicals [1] The environmental problems associated with petroleum-based products, coupled with ever-increased demand on crude oil have led to the perception that renewable alternatives should be seriously considered and developed. For example, it is estimated that within two decades fossil-based resources will not be enough to meet world demand. In this regard, biomass-based raw materials have attracted much interest and proved to be a feasible alternative [2,3,4]. Additionally, synthetic polymers are resistant to chemical, photochemical, and enzymatic degradation. This has led, *inter alia*, to an increasingly serious waste disposal problems resulting, e.g., in discouraging/banning the use of polyethylene bags in the supermarkets of several countries.

Polymers are currently employed in diverse sectors, including paint, food, cosmetic, car, and building industries. In most of these applications, biopolymers, particularly those from renewable sources such as cellulose, chitin/chitosan, and starch represent interesting alternatives, due to their structural versatility, ready biodegradability, and relatively low cost [5,6]. Undoubtedly, these eco-friendly polymers are an important contribution in the search for solutions for the waste-disposal problem; the reduction of CO_2_ emission; the development of biocompatible devices, and edible packing films [2,3,4]. In summary, derivatives of biopolymers are here to stay because of their compliance to the principles of green chemistry, in addition to their favorable properties and competitive cost.

## 3. Derivatization of Cellulose, Chitin/Chitosan, and Starch

### 3.1. Relevance of the Molecular Structures of Cellulose, Chitin/Chitosan, and Starch to Biopolymer Processing and Derivatization

**Figure 1:**
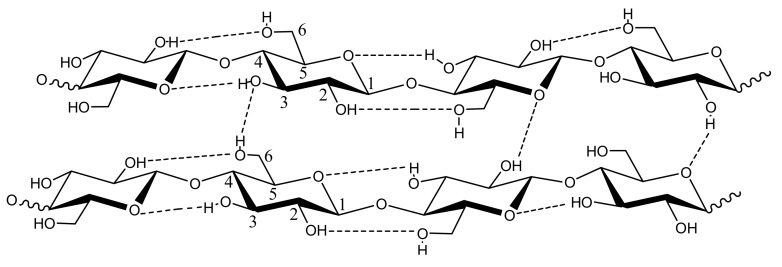
Intra- and intermolecular hydrogen bonds in cellulose. The anhydroglucose units, AGUs, are linked by 1,4-β-glycosidic bonds.

Figure 1 shows that the molecular structure of cellulose leads to extensive inter- and intra-molecular hydrogen bonding [7]. The consequence of this bonding, and van der Waals interactions, [8] is that cellulose chains align in a highly ordered state to form crystalline regions, whereas the less ordered segments constitute the amorphous part. The proportion of ordered to disordered regions (index of crystallinity, Ic) of cellulose varies considerably with its origin and the extent of treatment, both physical and chemical, to which the raw material, e.g., wood was submitted.

This structural feature bears on several aspects of the chemistry and applications of cellulose; we dwell here on cellulose processing and reactivity. For example, cellulose cannot be processed by the techniques most frequently employed for synthetic polymers, namely, injection molding and extrusion from the melt. The reason is that its temperature of melting presumably lies above the temperature of its thermal decomposition. Several commercial cellulose derivatives, in particular cellulose acetate, CA, and nitrate, are soluble, however, in common organic solvents, e.g., acetone, alcohol and chloroform, and can be extruded as fibers, films, rods and sheets. Since the AGU has three free OH groups (at C2, C3 and C6) it is possible, in principle, to obtain derivatives of any degree of substitution, DS, *directly* by adjusting the molar ratio (derivatizing agent)/AGU. In practice, however, this is not feasible, because: (i) the three hydroxyls have different reactivities both under heterogeneous [9], and homogeneous reaction conditions [10]. (ii) The accessibilities of the *same* hydroxyl group in the amorphous and crystalline regions are different [11]. Consequently, it is not feasible to obtain *uniformly substituted* cellulose derivative with DS, say of 1 to 2.5 directly, *i.e.*, by the (heterogeneous) reaction of a slurry of cellulose in the derivatizing reagent. The reason is that the products obtained will be heterogeneous, even if the (average) DS is achieved. The AGU’s of the amorphous regions will be more substituted than their counterparts in the crystalline regions. This heterogeneity may lead, for example, to serious solubility problems in solvents that are usually industrially employed, e.g., acetone [12].

**Figure 2:**
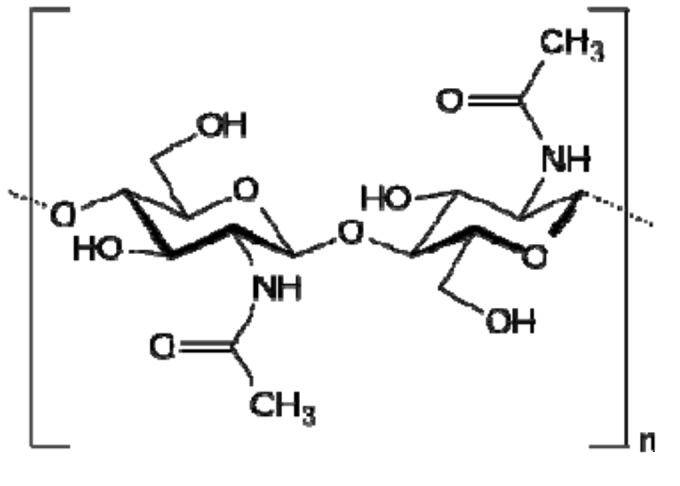
Molecular structure of chitin.

Chitin is a high molecular weight linear homopolymer of β-(1,4)*N*-acetyl glucosamine [13]. Similar to cellulose, chitin is a fibrous polymer whose structure is characterized by multiple hydrogen-bonding linkages. The network formed by that set of linkages confers high strength to chitin, see Figure 2.

**Figure 3:**
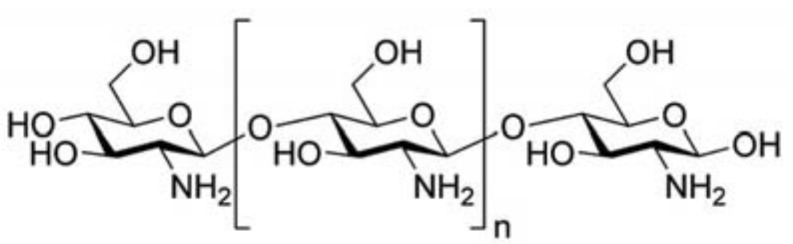
Molecular structure of chitosan.

Chitosan is obtained by deacetylation of chitin e.g., by a base. As this reaction is usually incomplete, chitosan is in fact a copolymer containing D-glucosamine and *N*-acetyl-D-glucosamine as monomers. The monomer are joined by β-(1,4) glycosidic linkage; see Figure 3.

Chitin and chitosan can be functionalized at two distinct functional groups, *viz*., OH and NH**_2_**. This has led to intense interest in a number of biotechnological applications that extend to pharmacy (in drug delivery, as hydrogels), food and nutrition, cosmetics, medicine (absorbable sutures, artificial skin, contact lenses, tissue regeneration), waste water processing, textile industry (sorption of dyes), paper industry (imparting wet strength to paper), photography, *etc*. [13,14].

**Figure 4:**
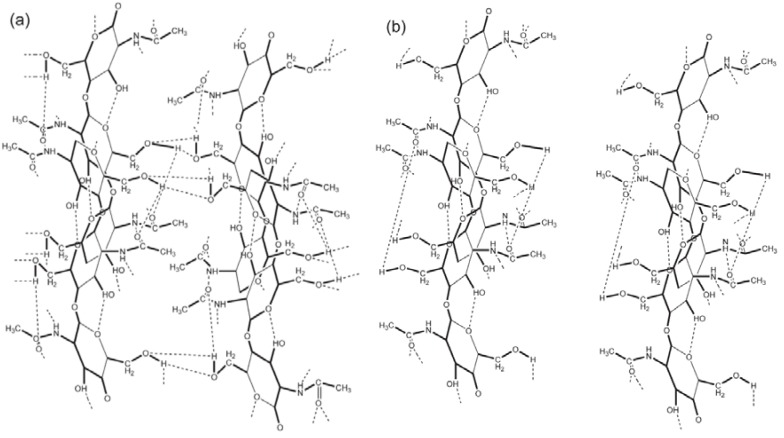
Characteristic hydrogen bonding patterns in (**a**) α-chitin; (**b**) β-chitin (reproduced from [18] with permission).

Chitin occurs in three different polymorphic forms (α, β and γ), the latter being a variant of the α-form that differs in packing and polarities of adjacent chains in successive sheets [15,16]. The capacity of solvents to solubilize chitin is dependent on the polymorph considered, which, in turn, varies with physiological role and tissue characteristics of the organism. In both α- and β-polymorphs, the chitin chains are organized in sheets tightly held by a large number of inter-sheet hydrogen bonds that form a tightly packed network. The α-chitin structure is highly crystalline, with both intra- and intermolecular hydrogen bonding (the latter between chains arranged in an antiparallel form) creating an intricate network that limits the access of solvent. Due to its more open structure, as shown in Figure 4, β-chitin is more susceptible than its α-counterpart to intracrystalline swelling. Note that the α-chitin polymorph can be converted into its β-counterpart by treatment with NaOH solution [15]. In general, however, chitin is a very intractable material due to its internal, highly hydrogen-bonded structure, being only soluble in concentrated acids or in solvent systems, e.g., LiCl/DMAC. Due to its ready protonation, chitosan dissolves in dilute acids, from which it can be extruded as gels and films [17].

**Figure 5:**
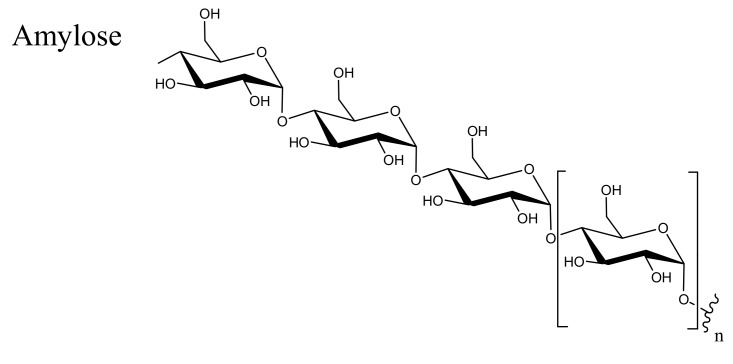

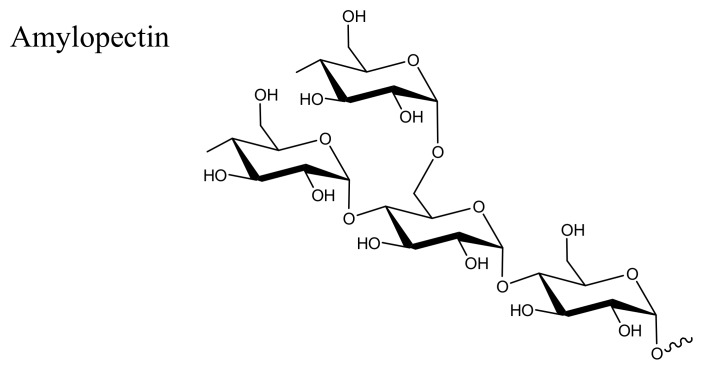
Molecular structures of amylose and amylopectin.

Starch consists of two types of biopolymers: amylose (linear and helical) and amylopectin (branched). Their structures are depicted in Figure 5. Amylose is formed by α(1→4) bound glucose molecules; amylopectin also presents α(1→4) linkages in its linear chain regions; however, it also shows branching points involving α(1→6) bonds, which occur at every 24 to 30 glucose units. Amylose represents *ca*. 25% of starch, the rest being amylopectin. These figures, however, are dependent on plant origin and also on soil conditions [2].

Starch gelation and retro gradation (a reaction that takes place in gelatinized *starch,* when amylose and amylopectin chains realign themselves, causing liquid to gel) is an important aspect of starch technology in many areas of application [2]. Functional characteristics of gels and of gelling process are markedly dependent on starch source and on relative amounts of amylopectin and amylose in starch grain.

Table 1 shows typical amylose and amylopectin contents of some starches [19,20,21]. The relevance of the ratio between the two components is that it bears on the DS of the derivatives. For example, starches with low amylose content exhibit higher DS on acetylation [22].

In summary, molecular structural features of three biopolymers have important consequences for their processing and derivatization. Some are not soluble in organic solvents; for products with intermediate DS values they cannot be derivatized directly and uniformly (in AGU and along the biopolymer backbone) by heterogeneous reactions; the accessibility of functional groups present depend on Ic, and on the type (primary or secondary) of the (OH) group; chitosan is interesting because it carries two functional groups (OH and NH**_2_**) having different nucleophilicity.

### 3.2. Principles of Polysaccharide Derivatization Under Heterogeneous and Homogeneous Reaction Conditions: Strong Electrolytes in Dipolar Aprotic Solvents

The production of cellulose esters and ethers by industrial processes, *i.e.*, under heterogeneous reaction conditions are well-established processes. Thanks to relatively recent developments (e.g., fast acetylation/fast hydrolysis process for CAs) these processes are cost-effective; there is no immediate need for major changes in industrial plants. For commodity products, e.g., CA and carboxymethyl cellulose (CMC), the properties are “adjusted” by blending several batches. Due to these aspects, derivatization under heterogeneous conditions faces limitations whenever a more rigid control of product characteristics, hence applications, e.g., in filters for hemodialysis where blood compatibility is an essential requirement [23]. The (unavoidable) decrease of DP during cellulose derivatization under heterogeneous conditions (e.g., due to acid- or base-catalyzed degradation) is, sometimes intentional, e.g., in order to decrease the viscosity of cellulose xanthate in the rayon production process. Blending of the products of several batches leads to products with acceptably reproducible characteristics/performance. A noticeable limitation is that the heterogeneous reaction is not employed commercially for the production of relatively hydrophobic esters, or ethers. These compounds are important because of their lower melting temperature (leading to less drastic extrusion conditions); higher solubility in common organic solvents, and compatibility in blends with relatively hydrophobic polymers. In fact, commercially available ester with the longest acyl group chain is cellulose butyrate. Another problem is connected with obtaining “one-pot” products with mixed substituents, e.g., acetate/butyrate derivatives. This is due to the intrinsic difficulty of controlling the reactivity of two competing reagents (e.g., acetic- and butyric anhydride) under heterogeneous conditions.

On the other end of spectrum is the homogeneous reaction scheme, HRS, in which biopolymer is dissolved in a non-derivatizing solvent, *i.e.*, one that causes dissolution without forming covalent bonds. This is followed by reaction with a derivatizing agent (acid anhydride; acyl chloride/base; alkyl halide/solid NaOH) to give the desired product. A recent interesting extension of HRS is that employed for obtaining ethers, by using ILs with basic counter-ion, because the reaction does not require an inorganic base in order to activate cellulose, hence is carried out under completely homogeneous conditions [24]. In principle, HRS is free of the consequences of the semi-crystalline structure of cellulose on reactivity because biopolymer chain is decrystallized upon solubilization [25]. Therefore, products are expected to be essentially regularly substituted, both within AGU and along the biopolymer backbone. Additional advantages of HRS include: little degradation of the starting polymer; high reproducibility; better control of reactions leading to the introduction of two functional groups (as in mixed esters) [26]. Whereas the relevance to industrial application of negligible cellulose degradation maybe open to question, HRS is definitely superior in terms of much better control of the product characteristics, hence performance. The latter fact is the impetus of continued intense interest in pursuing different aspects of this scheme.

#### 3.2.1. Derivatization of Cellulose Under Homogeneous Reaction Conditions

There are only a few solvents that dissolve cellulose physically, *i.e.*, without forming a covalent bond. These include in *N*-methylmorpholine *N*-oxide [27], alkaline solutions [28], and ionic liquids [7,29]. Most other molecular solvents cause swelling of cellulose to varying extents, but not complete dissolution. Nevertheless, disruption of these interactions can be readily achieved by using strong electrolytes, SEs, in dipolar aprotic solvents, DAS. Examples of SEs include LiCl and tetraalkylammonium fluoride hydrates (R_4_NF·xH_2_O). Examples of the DAS are *N,N*-dimethylacetamide, DMAC, *N*-methylpyrrolidin-2-one, and DMSO. Briefly, these electrolytes dissociate in the DAS employed, due to their high polarities and relative permittivity’s. A combination of biopolymer-solvent system interactions, including those with the unsolvated ions, and/or their complexes with DAS disrupt the hydrogen-bond network present, leading to biopolymer dissolution. The importance of components of solvent system and the structural characteristics of cellulose can be shown by the following results: (i) tetra (1-butyl) ammonium chloride and bromide are soluble in DMSO but do not dissolve cellulose [30]. (ii) in the same DAS, LiCl is more effective than LiBr; (iii) TBAF/DMSO dissolves cellulose at room temperature; the corresponding tetramethylammonium fluoride is ineffective; benzyltrimethyl-ammonium fluoride hydrate is only partially satisfactory [31], (iv) MCC dissolves in LiCl/DMAC more readily than fibrous celluloses; dissolution of the latter depend on their DP and Ic; cotton is frequently mercerized in order to facilitate its dissolution [32,33].

In recent years, LiCl/DMAC and tetrabutylammonium fluoride trihydrate (TBAF) have become popular solvent systems for dissolution of cellulose, chitin/chitosan and starch. The former system was first employed in order to dissolve polyamides and chitin [34,35,36,37,38]. Its use quickly spread, and the application to dissolve cellulose was reported for the first time almost concomitantly by McCormick [39] and Turbak [40]; the (TBAF) system has been developed thanks to the work of Heinze et al, *vide infra*. The mechanisms involved in biopolymer dissolution by these solvent systems will be discussed below in more detail.

Despite the advantages of HRS in terms of better product control, there is an obvious need to evaluate the environmental and economic aspects of this approach. Although this discussion is outside the scope of the present overview, we note that published toxicological data show that DMAC [41] and DMSO [42] are much safer solvents for derivatization than, e.g., dichloromethane [43] that is employed in industrial, *i.e.*, heterogeneous preparation of cellulose acetate [44]. Due to relatively high cost of the SE/DAS system, it is imperative that HRS is optimized in order to be competitive. For example, DMAC, unreacted acetic anhydride, and the produced acetic acid have been recovered, essentially pure, from the reaction mixture by fractional distillation under reduced pressure [32]. Although no attempt has been made to recover LiCl, it can be precipitated by addition of a suitable, less polar solvent. In principle, heating solutions of quaternary ammonium fluorides may lead to side reactions, e.g., Hofmann elimination [45]. Therefore understanding details of solvent-biopolymer interactions and physical state of biopolymer in solution, and recycling the components of solvent system are essential elements for commercial success of this process; some of these will be examined in the following sections.

##### 3.2.1.1. Strategies for Cellulose Activation: Solvent Exchange; Water Entrainment by Partial Solvent Distillation; Thermal Activation

Depending on the solvent system employed in order to dissolve cellulose, it is necessary to submit the biopolymer to an “activation” pretreatment step, before its dissolution is attempted. This is the case for LiCl/DMAC; R_4_NF/DMSO dissolve MCC and fibrous celluloses directly, *i.e.*, without prior activation. The objective of activation is to increase the diffusion of reagents into cellulose supra-molecular structure, by making the crystallite surfaces and the crystalline regions more accessible. This is achieved by inter- and intra-crystalline penetration of activating agent into cellulose, which disrupts strong, water-mediated hydrogen bonding between biopolymer chains [11,46]. The relevance of this step to the success of reaction is demonstrated by erratic results that are obtained if it is not carried out properly. The following results of cellulose acetylation with 50 wt% acetic anhydride in pyridine, at 30 °C, drive home the point (the figures refer to acetyl content): no activation, 8.8%; pre-treatment with chloroform/pyridine, 26.4%; same pre-treatment with ethanol/chloroform, 27.6% [47]. Therefore, we start by describing the different strategies that are employed for cellulose activation. Activation by treatment with a base, e.g., NaOH will not be considered in the present account because the base reacts with most of the derivatizing agents, in particular those that are employed for ester and ether formation. The three main methods employed are described below:

###### Activation by Solvent Exchange

Native or mercerized cellulose can be activated by a solvent exchange scheme, in which the biopolymer is first swollen with water; the latter is displaced by methanol, and then finally by the derivatizing DAS, e.g., DMAC [48,49,50]. For biopolymers of different structural characteristics, sufficient time should be permitted for the chains to be untangled. The larger the Ic and the molar mass of the sample, the longer is the time needed to obtain a clear solution (after addition of LiCl). This method is universal, applicable to all types of cellulose, including bacterial cellulose. It is, however, both laborious and expensive. For example, one day is needed for the activation of MCC, by using 25 mL of water; 64 mL of methanol, and 80 mL of DMAC/g cellulose. Its use is recommended where cellulose dissolution with almost no degradation is required.

###### Water Entrainment by Partial Solvent Distillation

Activation by distillation of a part of reaction solvent (*ca.* 25%) is based on the fact that at its boiling point, DAS has sufficiently high vapor pressure to cause extensive fiber swelling. [51,52,53,54]. This single-step method is simpler, faster than solvent exchange, and consumes less LiCl for biopolymer dissolution [55]. Two problems, however, are associated with this method: (i) It does not eliminate water completely, which leads to consumption of a part of acylating agent [56]; (ii) its use may lead to biopolymer degradation by two routes: The first involves the formation of furan structures by reaction of biopolymer with, *N,N*-dimethylacetoacetamide, CH**_3_**CO-CH**_2_**CON(CH**_3_**)**_2_**, a primary auto-condensation product of DMAC. Reaction of cellulose with this condensation product is slow, and is catalyzed, e.g., by carboxylic acid that is liberated during acylation by a carboxylic anhydride. A faster biopolymer degradation reaction involves *N,N*-dimethylketeniminium ion [CH_2_=C=N^+^(Me)_2_] that is formed by dehydration of enol tautomer of DMAC [CH_2_=C(OH)N(Me)_2_] This extremely reactive electrophile causes random chain cleavage, resulting in pronounced and rather fast changes in the molar mass distribution of cellulose [57].

###### Thermal Activation

Activation can be carried out by heating. Because this treatment may lead to biopolymer “hornification”, it is usually carried out under reduced pressure. In one procedure, a mixture of cellulose and LiCl is heated under reduced pressure until the water in cellulose is removed, followed by introduction of DMAC [58]. It is important that DAS is also introduced under reduced pressure; establishing atmospheric pressure before the heat-activated polymer is embedded by the solvent leads to erratic results, probably due to pressure-drop induced hornification. This method is simple, less time consuming, and does not cause biopolymer degradation [59].

##### 3.2.1.2. Mechanism of Cellulose Dissolution

**Figure 6:**
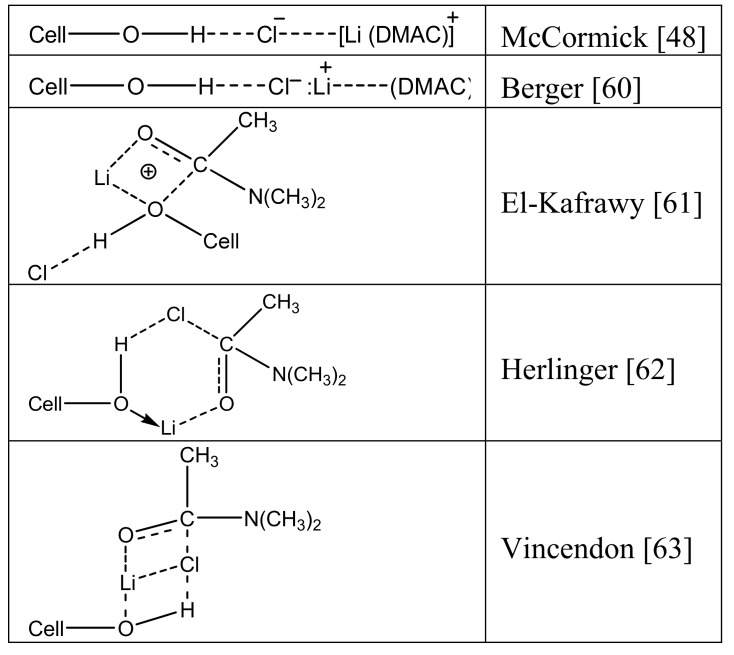
Proposed mechanisms of cellulose-LiCl/DMAC complexation.

Alternative models have been advanced in order to explain the mechanism of solubilization, some of which are summarized below, Figure 6 [48,60,61,62,63]. Most of these are based on the interactions between SE/DAS complex, its component simple- or complex ions (e.g., Li(DMAC)^+^ macro-cation) and the hydroxyl groups of cellulose [64,65,66,67].

**Scheme 1:**
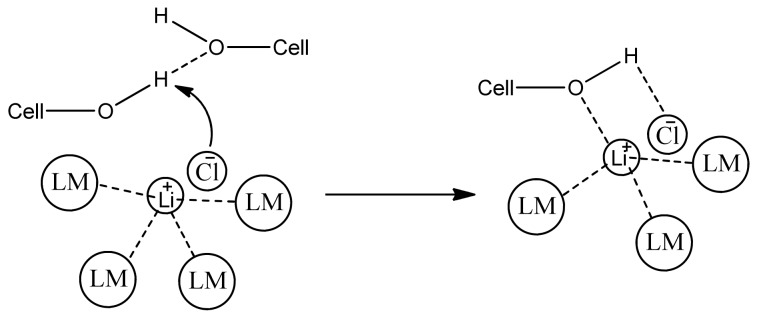
Proposed model for cellulose/LiCl/DMAC interaction leading to dissolution; LM refers to DMAC molecules solvating the Li^+^ ion.

The formation of these structures has been probed by NMR spectroscopy. For LiCl/DMAC [68], a decrease in ^7^Li chemical shifts and increase in its peak width at half-height was observed as a function of increasing cellulose concentration. In contrast, no variation in these NMR parameters was observed for LiCl/DMAC solutions in the absence of cellulose, as a function of increasing [LiCl]. Therefore, molecular environment of Li^+^ progressively changes as cellulose is added to the solution. The interaction presumably involves an exchange between one DMAC molecules in the inner coordination shell of Li^+^ with a cellulosic hydroxyl group, in a cooperative manner. In addition, the bulky LiCl/DMAC complex would penetrate into the cellulose chains, creating more inner space within 3D biopolymeric structure, thus contributing further to dissolution. This exchange model is shown in Scheme 1 [68].

The importance of Cl^−^·····H-O-Cell, interactions for cellulose dissolution in LiCl/DMAC has been corroborated by the study of solvatochromism in these solutions. The latter term refers to the effect of medium on the spectra, absorption or emission, of certain compounds (solvatochromic substances or probes) whose spectra are especially sensitive to the properties of the medium. These properties include “acidity”, “basicity”, dipolarity, and polarizability. The information on the properties of the medium is usually obtained from the dependence of solvatochromism (*i.e.*, the value of λ_max_ of the probe intra-molecular charge-transfer complex) on some experimental variable, e.g., concentration or solution temperature. These probes have been employed in order to investigate the properties of cellulose proper; DMAC, LiCl-DMAC; and cellulose/LiCl-DMAC solutions [69,70]. Thus high “acidity” of unsolvated Li^+^ was reduced in LiCl/DMAC solution indicating the formation of Li^+^(DMAC)_n_ macro-cation.

Whereas dissolution of cellulose in LiCl/DMAC has little effect on the overall polarity of DAS, the basicity of the medium was affected drastically, indicating strong Cl^−^ ·····H-O-Cell interactions. It was concluded that the basicity of the medium (due to both Cl^−^ and the C=O dipole of DMAC) contributes much more than the corresponding acidity (due essentially to free- and complexed Li^+^ ion) to cellulose solubilization. These results agree with previous conclusions on the mechanism of cellulose dissolution in DMAC/LiCl [67]. It is interesting to mention that cellulose dissolution in LiCl/DMSO requires decrystallization pretreatment e.g., by ball-milling or extensive swelling by a base [71].

**Figure 7:**
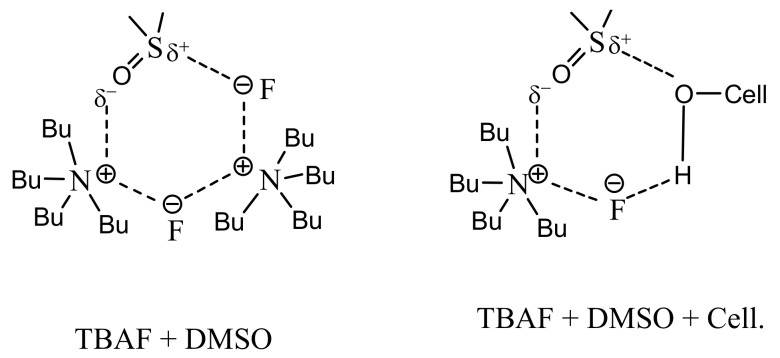
Simplified structures for the interaction of TBAF and DMSO, [7] and for cellulose solution in TBAF/DMSO.

The similarity between dissolution by LiCl/DMAC and TBAF/DMSO has been suggested, as shown in Figure 7. The six-membered “ring” that involves two TBAF molecules and one DMSO [7], or the structure in the presence of cellulose, where the biopolymer is shown to substitute one TBAF molecule (our representation) are clearly oversimplifications due to relatively large distance between (C_4_H_9_N^+^) and nucleophilic species in solution, including (F^−^) counter-ion, and oxygen atoms of the solvent (DMSO; distance R_4_N^+^·····O-solvent > 0.35 nm**)** [7,72].

Phase diagrams, rheology, and NMR (^19^F and ^1^H-NMR, chemical shifts and line widths) have been employed in order to investigate the effect of presence of water on MCC/LiCl-DMAC, and the interactions of cellulose with TBAF/DMSO.

**Figure 8:**
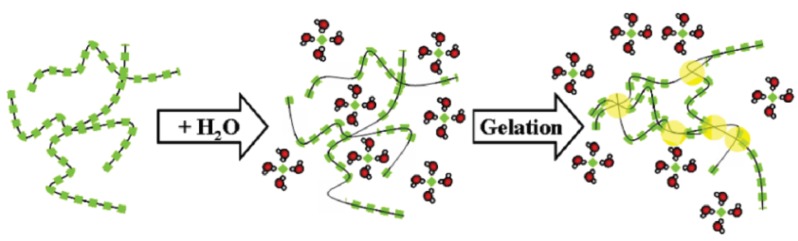
Schematic representation of the effect of water on solution of cellulose in TBAF/DMSO (reproduced from [74] with permission).

The former study has indicated that the maximum water content that can be present in the samples so that no cellulose precipitation- or liquid crystal formation occurs is always <3 wt%, even in the most concentrated DMAC/LiCl solutions. The amount of water still tolerable in the mixture is strongly dependent on the concentrations of cellulose and LiCl, being inversely proportional to biopolymer [73]. For solutions of cellulose in TBAF/DMSO, NMR results have indicated that the highly electronegative F^−^ ions act as hydrogen-bond acceptors of Cell-OH groups; this breaks the intermolecular hydrogen bonds between cellulosic chains, leading to dissolution of the biopolymer. Solubilization is enhanced by electrostatic repulsion between the negatively-charged cellulose chains, due to the condensation of F^−^. Addition of water solvates the fluoride ion, this leads to a decreases of cellulose solubility and, eventually, to solution gelation. This sequence of events is shown in Figure 8 [74].

The cellulose chains are covered with associated fluoride ions (depicted in green). Added water (depicted in red) solvates a fraction of the F^−^ ions that are associated with cellulose. The resulting desolvated biopolymer chains (depicted in yellow) associate, by combination of hydrogen-bonding and hydrophobic interactions [8], leading to subsequent precipitation of the biopolymer (reproduced from [74] with permission).

It is important to emphasize that the formation of clear, macroscopically homogeneous cellulose solutions in SEs/DAS does not necessarily mean that chains are molecularly dispersed. Rather they are present as aggregates- designated as “fringed micelles [75], whose aggregation numbers (e.g., 11 for MCC; 21 of mercerized-sisal; 40 for mercerized-cotton) depend on the structural properties of cellulose, its concentration, and the method of solution preparation. This aggregation decreases the accessibility of biopolymer, hence the efficiency of its derivatization [75].

The consequence of this aggregation is that the efficiency of the reaction, in terms of the ratio (derivatizing agent/AGU) that is required in order to achieve a targeted DS is rarely stoichiometric; employing excess reagent is the role. Table 2 summarizes the results on cellulose dissolution in SEs/DAS.

##### 3.2.1.3. Cellulose Derivatization

**Scheme 2:**
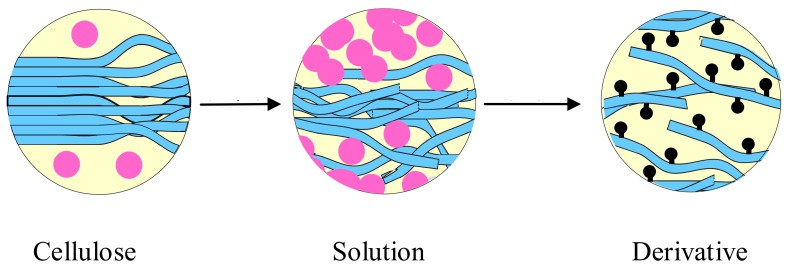
A schematic representation of derivatization by the HRS. Activation and Dissolution lead to the formation of solvated cellulose chains; these react with the derivatizing agent to produce a cellulose derivative.

A schematic representation of cellulose derivatization by the HRS is shown in Scheme 2.

**Scheme 3:**
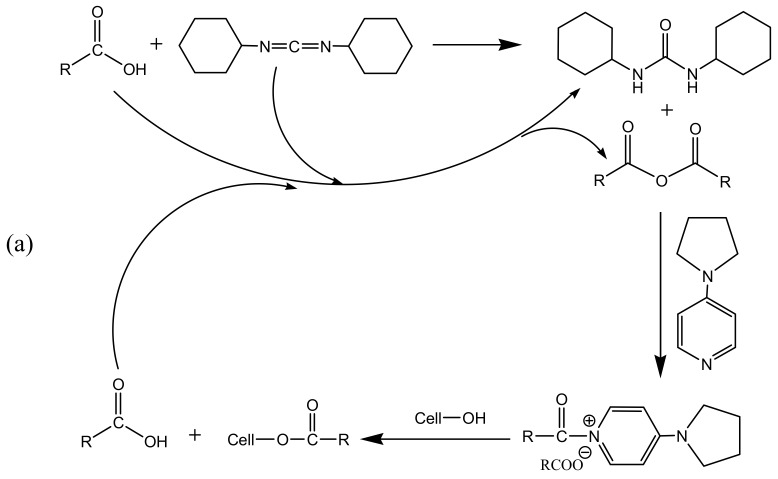

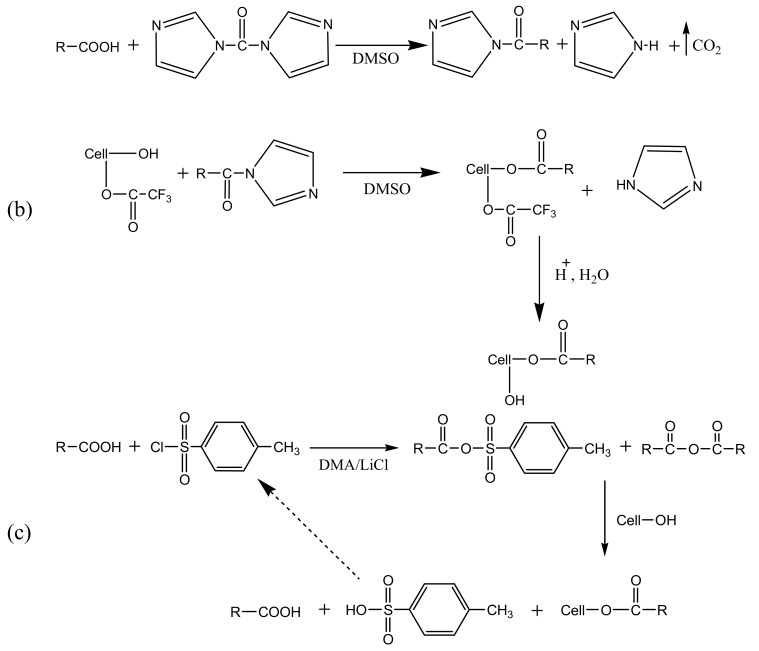
Schemes for the *in situ* activation of carboxylic acids. (**a**) shows activation by DCC; (**b**) shows activation by CDI, resulting in the formation of reactive *N*-acyl imidazole; (**c**) shows the formation of mixed anhydride between carboxylic- and toluene sulfonic acid.

In principle, derivatization of cellulose can be carried out by using either carboxylic acids proper, or their functional derivatives. The latter include: symmetric and asymmetric acid anhydrides and acyl chlorides in the absence, or presence of catalysts; diketenes; vinyl esters; lactones and lactams. Due to relatively low pKa of the hydroxyl groups of sugars (12.3 ± 0.3) [126] direct esterification with carboxylic acids is inefficient; these have to be activated *in situ* before use, as shown in Scheme 3a–c below [26]. One such acid-activating reagent is dicyclohexyl carbodiimide, DCC, either alone, or in combination with a powerful nucleophile, e.g., 4-pyrrolidinopyridine, Part A.

**Scheme 4:**
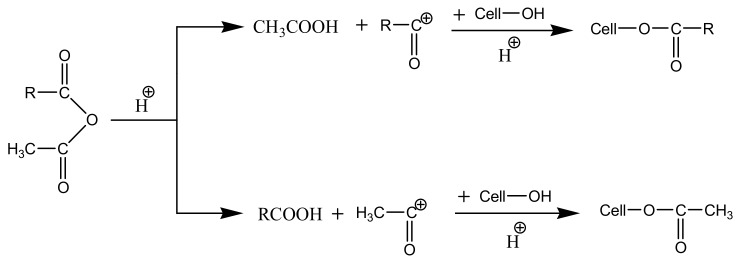
Formation of mixed anhydride of acetic- and fatty carboxylic acid.

First, acid anhydride is produced by the reaction of free acid with DCC. Nucleophilic attack by 4-pyrrolidinonepyridine on the anhydride results in the corresponding, highly reactive, acylpyridinium carboxylate; this leads to formation of cellulose ester, plus a carboxylate anion. The latter undergoes a DCC-mediated condensation with a fresh molecule of acid to produce another molecule of anhydride. *N,N*-Carbonyldiimidazole (CDI), may substitute DCC for acid activation, the acylating agent is *N*-acyl imidazole that readily reacts with cellulose to give ester and regenerate imidazole, part B. In another variant, activation is carried out by TsCl/pyridine. As shown, an asymmetric carboxylic-sulfonic acid anhydride is formed, but cellulose attack occurs on the C=O group, since nucleophilic attack on sulfur is slow, and the tosylate moiety is a much better leaving group than the carboxylate group. When the leaving abilities of both groups of the asymmetric anhydride are comparable, mixed esters are obtained. For example, cellulose esters of long-chain fatty acids, e.g., dodecanoate to eicosanoate have been prepared in LiCl/DMAC with this activation method, with almost complete functionalization, DS 2.8–2.9 [127]. The (mineral) acid-catalyzed formation of mixed acetic-carboxylic anhydride has been employed in order to synthesize mixed esters of acetic and fatty acids, according to scheme shown in Scheme 4 [128,129].

**Scheme 5:**
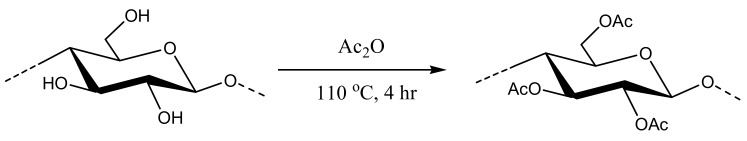
Schematic representation of the use of acetic anhydride as a derivatizing agent for cellulose.

The same approach has been employed for obtaining carboxylate-phosphonate mixed esters by the reaction of cellulose with carboxylic-phosphonic mixed anhydride [130]. Similar to other esterification reactions, there is large preference for tosylation at C6 position of AGU, and all accessible tosyl celluloses (up to DS = 2.3) are soluble in DMSO [131]. Symmetric carboxylic acid anhydrides are reactive enough to transform cellulose into its esters; see Scheme 5. This simple esterification reaction has furnished important information about structure/reactivity relationships in cellulose chemistry.

**Figure 9:**
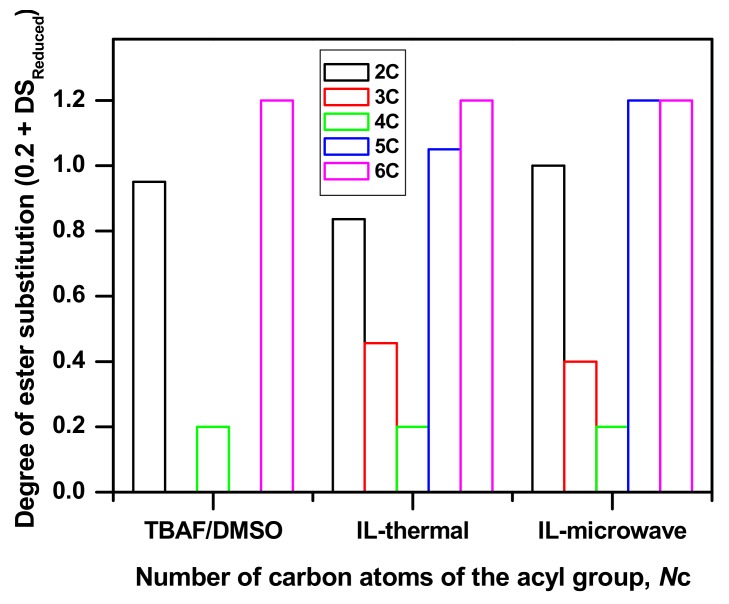
Dependence of DS_Reduced_ on *N*c in different solvents, under convection- and microwave heating. The DS are: 0.79, 0.38, and 2.40 (butyrate) 1.07, 2.72 and 2.90 (hexanoate), respectively.

Thus in the simultaneous reaction of cellulose with mixtures of acetic-, propionic-, and butyric anhydride, the DS_Acetate_ is usually larger than DS_Propionate_ or DS_Butyrate_ because of the higher electrophilicity of the acyl group-, and smaller volume of the first anhydride [25]. The efficiency of acetylation of MCC; mercerized cotton linters; mercerized sisal, as expressed by the dependence of DS on (RCO)_2_O/AGU is described by the following exponential decay equation:

[DS = DS_o_ + Ae^−[(RCO)2O/AGU)/**B**]^]

where (A) and (B) are regression coefficients. Values of (B) were found to correlate linearly with the aggregation number, N_agg_, of dissolved cellulose chains, (B) = 1.709 + 0.034 N_agg_. This result quantifies the dependence of cellulose accessibility, hence reactivity on its state of aggregation [75] For the same cellulose, under distinct reaction conditions, the dependence of DS on the number of carbon atoms of the acyl group of anhydride, *Nc*, is not linear; it decreases on going from acetic to butyric anhydride, then increases for pentanoic- and hexanoic anhydride, as shown in Figure 9. In the latter we have employed, for convenience, the following reduced degree of substitution:

[DS_Reduced_ = (DS_Carboxyate_ − DS_Butyrate_)/(DS_Hexanoate_ − DS_Butyrate_)]

This dependence, is not related to the solvent employed (SE/DAS or ionic liquid) or the method of heating, conventional (*i.e.*, by convection) or microwave. This is due to a complex dependence of the ΔH^≠^ and TΔS^≠^ terms on *Nc* [10].

Cellulose esterification with anhydrides is catalyzed by nucleophiles, in particular imidazole, pyridine, and 4-(*N,N*-dimethylamino) pyridine, with a large decrease in reaction time. The reactive species is the *N*-acyl derivative of the tertiary amine. A recent kinetic study on acylation in LiCl/DMAC has indicated that this rate enhancement, relative to the uncatalyzed reaction, is due to smaller enthalpy, and larger (*i.e.*, less negative) entropy of activation [132]. 

**Scheme 6:**
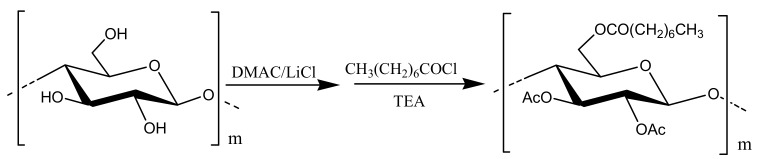
Schematic representation of acylation by carboxylic acid chloride/tertiary amine as a derivatizing agent.

Derivatization by acyl chloride/tertiary amine is shown in Scheme 6; base is employed in order to scavenge the liberated HCl, the results are similar to the reaction with acid anhydrides [133].

**Scheme 7:**
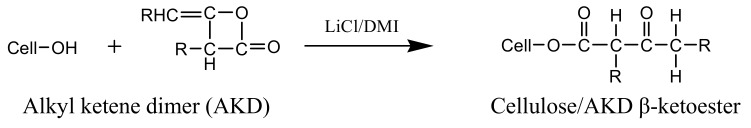
Representative scheme for the reaction of cellulose with alkylketene dimers. The produced β-ketoesters form enolates that can be employed in cross-linking of cellulose chain.

The reaction scheme with alkyl ketene dimers is shown in Scheme 7. Mixed acetoacetic/carboxylic esters have also been synthesized. Having a relatively acidic -methylene group, these β-ketoesters can be cross-linked to produce coatings with excellent solvent resistance [134,135,136,137].

Vinyl esters: e.g., vinyl acetate, benzoate and laurate have been employed in order to obtain cellulose esters in TBAF/DMSO. This is a (reversible) trans- esterification reaction. Its efficiency is based on the fact that one of the products, vinyl alcohol readily tautomerize to (volatile) acetaldehyde, thus driving the equilibrium to products [138].

**Scheme 8:**
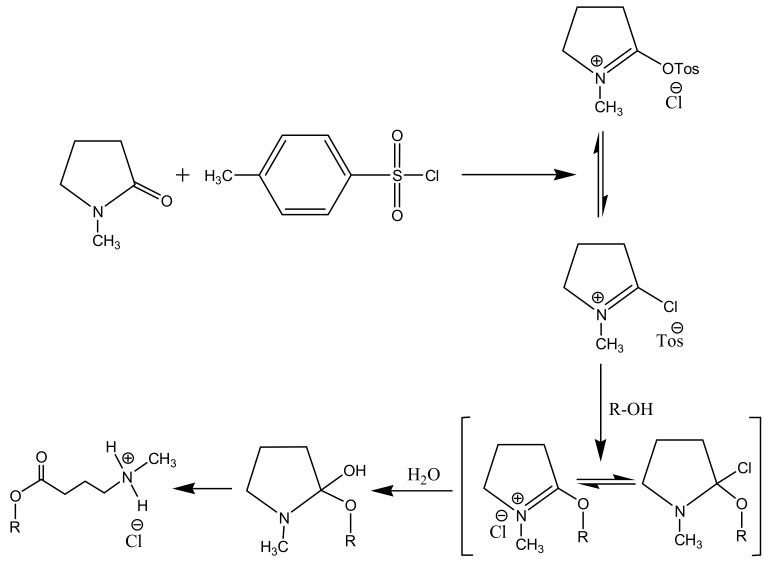
Schematic representation of the conversion of cellulose (ROH) into cationic ester by the reaction with *N*-methyl-2-pyrrolidinone.

Esters with a cationic charge have been synthesized by the reaction of a lactam (*N*-methyl-2-pyrrolidinone; ε-caprolactam; *N*-methyl-2-piperidone) with cellulose in the presence of TsCl, according to Scheme 8, where R-OH refers to cellulose [139]. Similar strategies have been employed for the synthesis of cellulose esters in R_4_NF·xH_2_O/DMSO solutions. This includes the reaction of cellulose with activated carboxylic acids [140], acid anhydrides and vinyl esters, [141,142], carboxylic acid anhydride catalyzed by a diazole or triazole [143,144]. This solvent system is interesting because: (i) It dissolves cellulose without prior activation; (ii) The efficiency as cellulose solvents depends on the molecular structure of the SE [32], (iii) The SE water of hydration leads to side reactions.

**Scheme 9:**
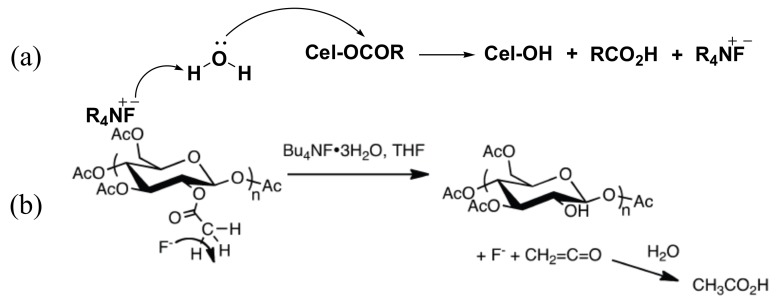
Suggested mechanisms for ester hydrolysis by TAAF in DMSO [142,145].

One of these is hydrolysis of the acylating agent. That is, relatively large molar ratios [anhydride]/[AGU] are usually employed. In this regard, the tetraallylammonium fluoride represents an interesting alternative to TBAF·3H**_2_**O because the former is obtained as monohydrate [142]. The second documented side reaction is hydrolysis of the produced ester either by a general-base catalyzed reaction [142], or via proton elimination followed by the formation of ketene. In fact, this hydrolysis reaction is regioselective, showing substantial selectivity for the removal of acyl groups at C-2 and C-3 positions, affording cellulose-6-*O*-esters with high regioselectivity by one-step reaction, without the use of protecting groups, see Scheme 9 [145].

The fluoride ion is acting either as a general base for water attack on the ester acyl group, part (a) [142], or abstracts a hydrogen from the acetate moiety, leading to formation of a good leaving group, ketene, part (b) (reproduced from [145] with permission).

Finally, the formation of acyl fluoride (RCOF) by the reaction between TAAF and acetic- or hexanoic anhydride (CH_2_=CH-CH_2_)_4_N^+^^−^F + (RCO)_2_O→RCOF + (CH_2_=CH-CH_2_)_4_N^+^^−^OCR) has been demonstrated by FTIR. That is, a part of cellulose derivatization probably proceeds by the reaction of cellulose and acyl fluoride [142].

**Scheme 10:**
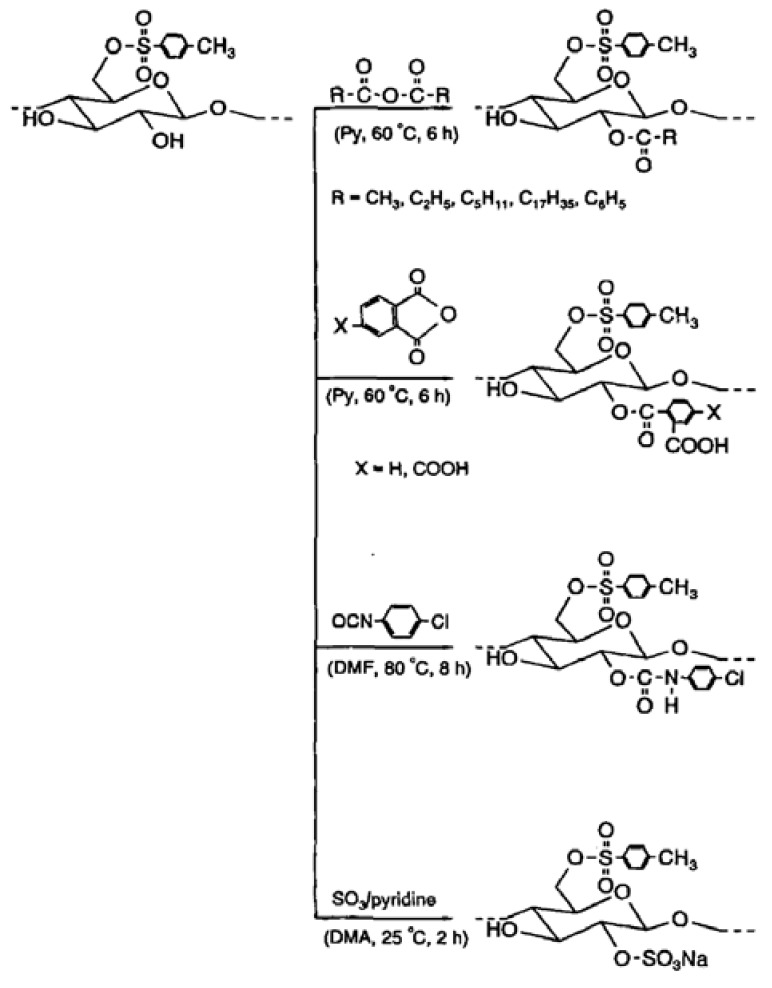
Use of tosylate moiety as a bulky group for C6-OH position of cellulose, leading to regioselective reaction at secondary hydroxyl groups (reproduced from [146] with permission).

Obtaining cellulose esters of other acids, e.g., tosylate; brosylate; mesylate; triflate is important *per se*, and because these moieties are employed for synthesis of cellulose derivatives with some control over regioselectivity. One such application involves their use as bulky groups, in particular at C6-OH position; this permits derivatization at C2-OH and C3-OH positions, see Scheme 10.

**Scheme 11:**
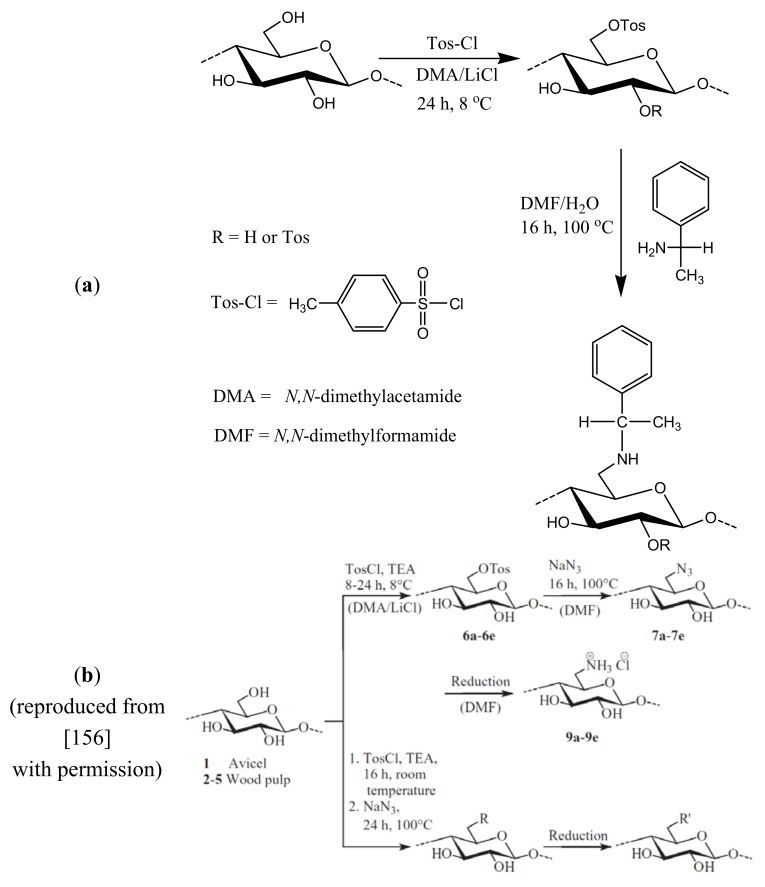

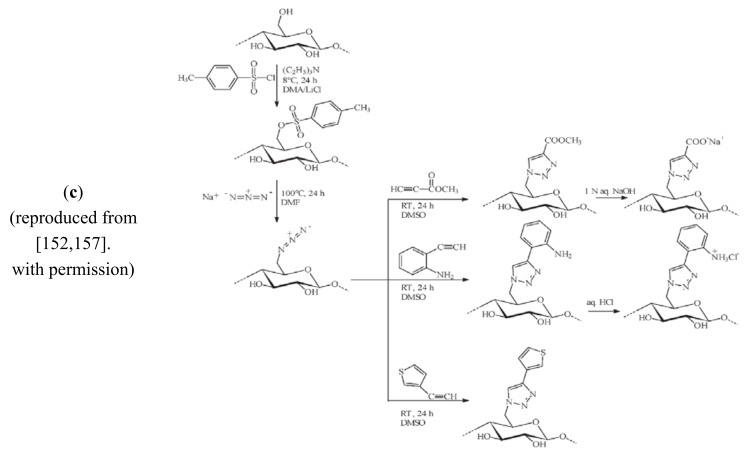
Synthesis of cellulose tosylate and its transformation into further products by S**_N_** reactions. (**a**) Transformation into cellulose deoxyamine. If required, the latter group can be quaternized to give cationic cellulose derivative [155]. The cellulosedeoxy azide can be converted into amines by reduction, part (**b**) [156], or into heterocyclic rings by click chemistry, part (**c**) [157].

They are also good leaving groups, so that they can be substituted by S_N_ reactions to produce cellulose deoxy derivatives [130,147]. In fact, this reaction usually occurs during tosylation by TsCl, the ratio of cellulose tosylate/deoxychlorocellulose is calculated from (Cl and S) elemental analysis [148]. The most extensively studied derivative of this series is the tosylate. It is carried out by reacting dissolved cellulose with tosyl chloride in the presence of TEA at low temperature (5–10 ^ο^C) for several hours, followed by ester precipitation and purification. Scheme 11 shows some examples of further S**_N_** reactions of cellulose tosylates.

**Scheme 12:**
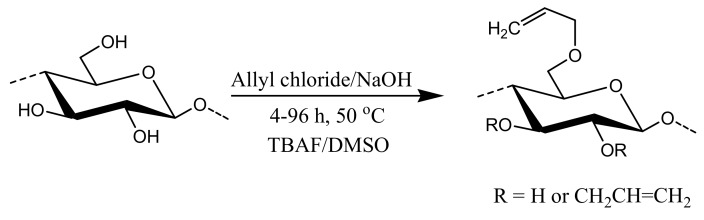
Allylation of cellulose dissolved in TBAF/DMSO.

The etherification of cellulose in LiCl/DMAC and TBAF/DMSO, even with reactive halides, e.g., allyl- and benzyl bromide is slow and requires long reaction time [149,150,151]. Therefore, an alkali is employed in order to activate cellulose, Scheme 12. An interesting procedure, employed for both cellulose and starch, is so called “induced phase separation”. This involves addition of finely divided, dry NaOH or KOH (usually obtained by employing with ultra Turrax mixers) to the cellulose/LiCl-DMAC solution, leading to the formation of cellulose II or starch reactive gels on the solid particle/solution interface; this enhanced reactivity leads to products with relatively high DS (e.g., 2.2 for CMC) [152,153,154].

**Scheme 13:**
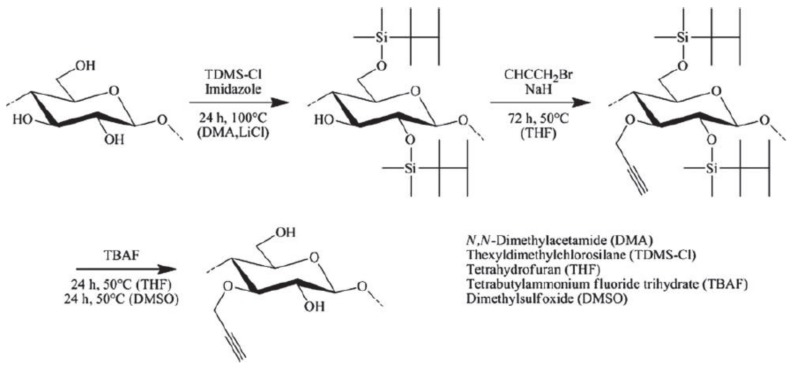
Using thexyldimethylsilyl moieties as protecting groups in the regioselective synthesis of cellulose ethers (reproduced from [158], with permission).

Another activation procedure involves imidazole, as shown in the synthesis of 3-*O*-propargyl cellulose by using thexyldimethylsilyl moieties as protecting groups, Scheme 13 [158,159]. It is worth mentioning that products with “mixed” functional groups have been synthesized in these solvents, e.g., ethers; [160,161].

Cellulose derivatizing agents and conditions most usually employed, as well as main techniques used, are summarized in Table 3. For convenience, we have organized the derivative in the order: Esters of carboxylic- and sulfonic acids; nonionic and ionic ethers, and miscellaneous derivatives.

#### 3.2.2. Dissolution and Derivatization of Chitin/Chitosan and Starch

**Scheme 14:**
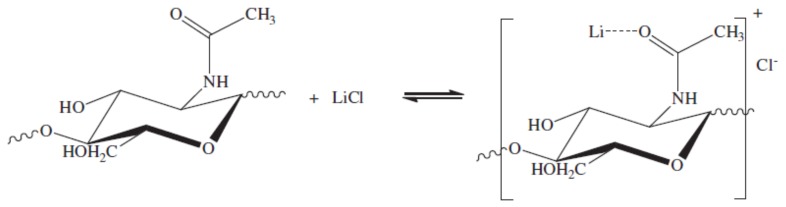
Model for chitin dissolution with LiCl (reproduced from [13] with permission).

Similar to cellulose, chitin dissolves in SE/DAS, allowing its derivatization. Although chitosan is soluble in aqueous acids, e.g., acetic acid, and starch can be dissolved DAS without the presence of SE, both biopolymers are included in our discussion of the SE/DAS solvent. The reason is that aqueous acid is not an appropriate medium for the derivatization of chitosan, due to hydrolysis of the derivatizing agent. Additionally, the use of SE/DAS accelerates the dissolution of starch. The mechanisms involved in their dissolution are essentially similar to those shown for cellulose (item 3.2.1.2); see Scheme 14, except that the activation pre-treatment is unnecessary [12]. Starch water solubility may limit the development of starch-based materials for applications where some hydrophobic character of the end-product is desired [162], as for instance in the development of edible packing films, in which water resistance is expected [163]. Some properties of interest can be introduced, such as thermo plasticity for instance, when starch is submitted to appropriate modifying reactions [162].

**Scheme 15:**
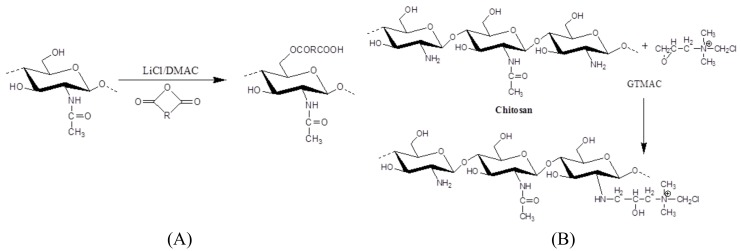
Route to the synthesis of chitin derivatives (**A**); Preparation of water-soluble chitosan derivatives (**B**).

**Scheme 16:**
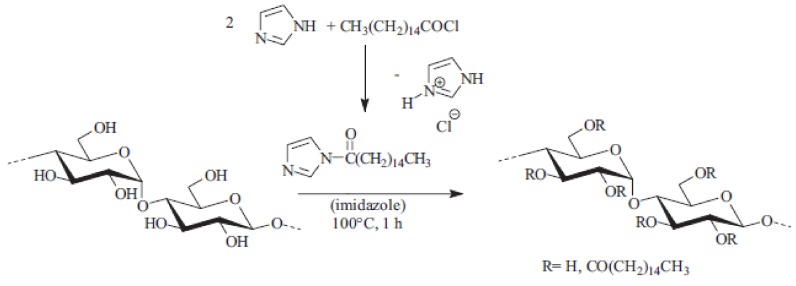
Reaction scheme for starch acylation in molten imidazole, by using carboxylic acid imidazolide obtained *in situ* (reproduced from [199] with permission).

Table 4 summarizes dissolution and derivatization conditions for starch and chitin/chitosan. Scheme 15 and Scheme 16 show examples of the derivatization of chitin/chitosan, and starch, respectively. For convenience, we have separated the published data on chitin/chitosan from those on starch.

**Scheme 17:**
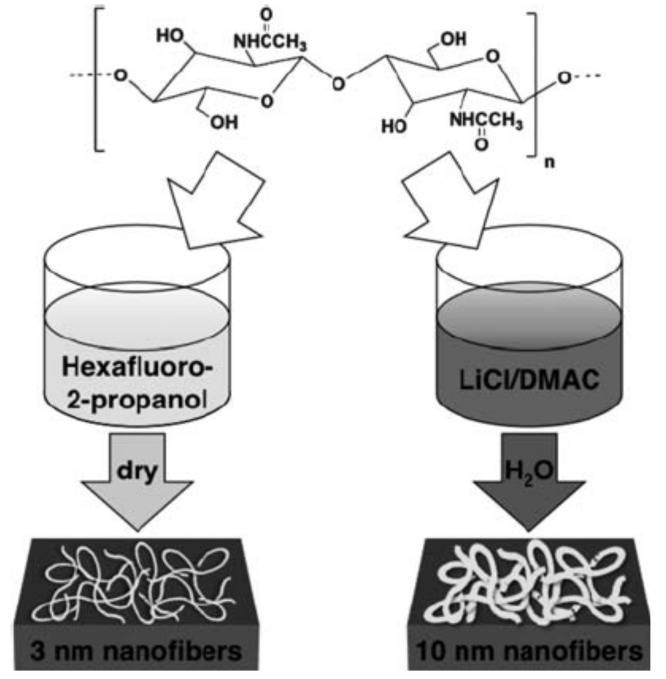
Schematic representation of the preparation of chitin nanofibers (reproduced from [201] with permission).

The LiCl/DMAC solvent system has been used more recently in a variety of applications of (bio) technological relevance. One interesting example is its use in a process for fabrication of biogenic chitin nanofibers. The self-assembly process of dissolved chitin is initiated with the addition of water, producing fine nanofibers (of crystalline α-chitin) of *ca.* 10 nm diameter. Hexafluoro-2-propanol can be used instead of LiCl/DMAC and similar results are obtained when the solvent is evaporated, see Scheme 17.

## 4. Concluding Remarks

The HRS offers interesting opportunities for cellulose chemistry. New, elegant synthetic schemes have been devised in order to control important aspects of biopolymer derivatization, in particular DS of the products and regioselectivity of substitution. The possibilities will continue to be explored, in particular with regard to new solvent systems and synthetic strategies that are consistent with principles of green chemistry. Process optimization calls for extensive kinetic data that should result in economy of time, power consumption, and a decrease of side reactions. Heating by microwave irradiation may prove a valuable tool to enhance reaction efficiency (higher DS in shorter reaction times). Economy of reagents dictates the use of stoichiometric reagent/AGU ratios, where possible.

In conclusion, the perspective for the HRS scheme is bright because it may be employed for obtaining specialty products, where the particularities of polymer structure and the consistency of product properties are central to performance. Examples include nano-composites; “smart” polymers that respond reversibly to external stimuli, and bio-compatible polymeric materials.

## Figures and Tables

**Table 1:** Typical amylose and amylopectin contents of starch from various crops.

Starch Source	% Amylose	% Amylopectin
Rice (Japonica)	17.5	82.5
Wheat (Asw)	21.7	78.3
Barley (Bomi)	27.5	72.5
Maize (Normal)	21.5	78.5
Maize (Hylon 7)	58.6	41.4
Water Chestnut	23.3	76.7
Chestnut	19.6	80.4
Sago	24.3	75.7
Lotus root	15.9	84.1
Kuzu root	21	79
Sweet Potato	18.9	81.1
Yam	22	78
Lentil	29–45	71–54
Tapioca	16.7	83.3
Arrowroot	25.6	74.4
Edible Canna	22.2	77.8
Potato	21	79
Waxy Maize	<1	>99
Corn	24–28	75
Waxy Corn	0	100

**Table 2:** Dissolution of cellulose from different sources in strong electrolytes/dipolar aprotic solvents.

Entry	Polysaccharide; DP; Ic	Dissolution solvent system	Dissolution conditions (temperature, heating time)	Techniques employed to study dissolution	Reference
1	MCC	5–8% LiCl/DMAC	150 °C, 2 h	GPC	[76]
2	Cotton Linters; 1776; 0.50 Sulphite pulp; 728; 0.42	3.5–8% LiCl/DMAC; EWNN	2–35 min; RT	SEM; TEM	[49]
3	Kraft pulps	8% LiCl/DMAC	4 °C; 5 d	^13^C-CP/NMR	[77]
4	MCC; 280 Sulphite pulp; 1020	7.5% LiCl/DMAC	130 °C; 1 h	Solvatochromic technique, UV/VIS	[64]
5	MCC; 155; 0.81 Bagasse; 780; 0.82	8.3% LiCl/DMAC	155 °C; 1 h	FTIR, X-Ray, SEM	[56]
6	MCC; 285 Buckeye; 1360	1–9% LiCl/DMAC	RT; overnight	LS	[78]
7	Cotton linters	8% LiCl/DMAC	<60 °C; RT	Viscometry	[79]
8	MCC; 440	5% LiCl/DMAC	100 °C; overnight	Fiber spinning method	[80]
9	Wood cellulose	8% LiCl/DMAC	80 °C; 4 h	SEC, MALLS	[81]
10	Cotton fiber	11% LiCl/DMAC	RT; overnight	SEC	[82]
11	Sulfite pulp	1–9% LiCl/DMAC	25 °C; 3 d	Phase diagram	[73]
12	Sulfite pulp	8% LiCl/DMAC	RT	WAXD, SAXS	[83]
13	Dissolving pulp	8% LiCl/DMAC	RT; few days	LS, SAXS	[84]
14	Whatman No.1 paper	8% LiCl/DMAC	RT; 15 h	SEC	[85]
15	Tunicate cellulose	8% LiCl/DMI	RT 6–9 months	LS, Viscometry	[86]
16	Whatman No.1 paper	8% LiCl/DMAC	RT; 24 h	SEC, MALS,DRI	[87]
17	MCC; 126; 0.83 Cotton linter; 400; 0.80 Sisal; 642; 0.67	7.4% LiCl/DMAC	150 °C; 1.5 h	X-Ray Diffraction, SEM,	[25]
19	Cotton linter	8% LiCl/DMAC	RT; 1 month	SLS	[88]
20	MCC	8% LiCl/DMAC	20 °C; 1, 4, 8, 48 h	WAXS, ^13^C-NMR	[89]
21	Fibrous cellulose 390	7% LiCl/DMAC	80 °C; 0.75 h	WAXD, FTIR	[90]
23	MCC; 332	7.5% LiCl/DMAC; 3.5% TBAF/DMSO	130 °C; 2 h 80 °C; 2 h	FTIR, ^1^H,^13^C-NMR	[31]
24	Soft wood sulfite pulp	8% LiCl/DMAC	RT; few h	SAXS, FTIR, ^13^C-NMR	[91]
25	Amorphous cellulose	1.5% LiCl/DMAC	160 °C; 20 °C, overnight	SAXS, AFM,	[92]
26	MCC	10% LiCl/DMAC	150 °C; 2 h, 150 °C; 3 min, microwave	^1^H-NMR, DLS, Viscometry	[93]
27	MCC; 163	8% LiCl/DMAC	RT; 3 min	WAXS, Density measurement	[94]
28	Bacterial cellulose	8% LiCl/DMAC	RT; 10, 15, 20, 40, 60 min.	X-Ray, SEM	[95]
29	MCC; 210–270	1% TBAF/DMSO	60 °C; 20 min,	^19^F,^1^H-NMR	[74]
30	Beach and spruce milled wood	6% LiCl/DMSO; 6% LiCl/DMAC	RT; 2 h	X-Ray diffraction	[96]
31	Cotton pulp; 4500	8% LiCl/DMAC	155 °C; 1 h	SEM	[97]
32	Cotton	8% LiCl/DMAC	155 °C; 1 h	SEM, UV-vis, X-Ray	[98]
33	Cotton	14% TBAF/DMSO	60 °C	^1^H-NMR, FTIR	[99]
34	MCC	9% LiCl/DMAC	RT; 1 h	X-Ray, SEM, ATR-FTIR,	[100]
35	MCC; 332	10% TBAF/DMSO	60 °C; 1 h	^19^F, ^1^H-NMR	[101]
36	Beech, eucalyptus	8% LiCl/DMI	RT; 3–4 d	SEC, LS	[102]
37	Cotton	9% LiCl/DMAC	155 °C; 4 h	Laser Doppler vibrometer	[103]
38	MCC	9% LiCl/DMAC	-------	SEC, SEM	[104]
39	Sisal; 642; 0.67 Cotton linters, 400; 0.80	8.3% LiCl/DMAC	150 °C; 1.5 h	X-Ray, Viscometry	[105]
40	Canola straw	8% LiCl/DMAC	RT; 5–120 min	X-Ray	[106]
41	Kraft pulp	8% LiCl/DMAC; 16.25% TBAF/DMSO	4 °C; 5 d	SEC, ^13^C-NMR	[107]
42	Cellulose membrane	8.1% LiCl/DMAC	100 °C; 6 h	HPLC	[108]
43	Cotton linters Softwood kraft pulp	9% LiCl/DMAC	40 °C; 0.5–120 h	GPC, SEM	[109]
44	Cellulose powder	10% LiCl/DMAC	100 °C; 7.5 h	SAXS, SEM	[110]
45	Kraft bleached pulp	8.5% LiCl/DMAC; 8.5% LiCl/NMP	-------	^13^C-NMR	[69]
46	Cellulose	5–10% LiCl/DMAC	-------	^1^H,^13^C, ^7^Li-NMR	[111]
47	Avicel; 170	LiCl/DMAC	RT; 1 h	^7^Li-NMR	[68]
48	Regenerated cellulose; 174	5–10% LiCl/DMSO	RT; 24 h	FTIR, ^13^C-NMR	[112]
50	oligosaccharides	0.01% LiCl/DMAC	100 °C; 1 h	ESI-TS, FAB	[113]
54	Wood cellulose	10% LiCl/DMAC	RT; 6 h	FTIR	[114]
55	MCC; 280	9% LiCl/DMAC	80 °C; 1.5 h	FTIR, WAXD, SEM	[115]
56	MCC; M-cotton; M-sisal	6% LiCl/DMAC	110 °C; 4 h	Viscometry, SLS, ^1^H-NMR	[71]
57	Sulfite softwood	8% LiCl/DMAC	35–40 °C; 1 day	SEC	[116]
58	Beech sulfite pulp	9% LiCl/DMAC	85–125 °C	GPC	[117]
59	Sulfite pulp; 1500	8% LiCl/DMAC	RT; 2 d	SEC	[118]
60	pulp	9% LiCl/DMAC	85–125 °C	^1^H-NMR	[119]
61	Cotton linter	8% LiCl/DMAC	RT; 1 month	SLS, DLS	[120]
62	Cotton cellulose	8% LiCl/DMAC	RT; 2–3 months	SLS, DLS	[121]
63	Dissolving pulp	8% LiCl/DMAC	RT; few days	SAXS	[122]
64	MCC; 280	9% LiCl/DMAC	150 °C; 1 h	SEM	[123]
65	Cotton linters	5.3% LiCl/DMAC	80 °C; 1 h	Viscometry	[124]
66	Hard wood kraft pulp	6–10% LiCl/DMAC	4 °C; 5 d	SEC	[125]

**Table 3:** Agents and conditions for cellulose derivatization and main study techniques.

Entry	Cellulose type; DP; Ic	Derivatizing agent	Reaction conditions: Ratio derivatizing agent/AGU; Temp. °C; Reaction time, h	DS range	Solvent used	Study Techniques	Reference
***Esters of carboxylic- and sulfonic acids ***							
1	MCC, hardwood pulp	Diketene; Butyric anhydride	3:1; 110 °C; 40 min.	0.90, 0.30	5% LiCl/DMAC, DMSO, NMP	GPC, DSC, ^1^H-NMR, ^13^C-NMR	[136]
2	MCC	Unsat. Carboxylic acids/DCC; acid anhydr./DMAP	25 °C; 48 h	0.25–0.55	9% LiCl/DMAC	FTIR, ^1^H-NMR	[164]
3	MCC; 0.79	Ac_2_O	1–9:1; 110 °C; 4 h	0.9–2.8	6% LiCl/DMAC	X-Ray, ^13^C-NMR	[58]
4	cellulose	Fatty acid chlorides, Ac_2_O	2:1; 130 °C; 5 h	2.9–3.0	9% LiCl/DMAC	^1^H-NMR, GC-MS,	[128]
5	MCC; 260	Acyl chlorides, TsCl	5:1; 80 °C; 2 h	2.96	7.5% LiCl/DMAC	^1^H-NMR, FTIR	[138]
6	Avicel; 330, sisal; 650	Ac_2_O; stearic anhyd; vinyl acetate	11:1; 60 °C; 3 h	1.20	11% TBAF/ DMSO	^1^H-NMR, HPLC	[165]
7	Cotton linter; 440	Ac_2_O	2–13:1, 60 °C; 3–24 h	0.43–2.77	9% TBAF/ DMSO	FTIR, ^1^H-NMR	[141]
8	MCC; 260	Ac_2_O; stearic acid; adamantane carboxylic acids; CDI	3:1; 80 °C; 24 h	0.5–1.9	10% TBAF/ DMSO	FTIR, ^1^H-NMR	[143]
9	MCC; 280	Carboxylic acids; CDI	5:1:5; 60 °C; 24 h,	2.5	7.5% LiCl/DMAC, 10% TBAF/DMSO	^1^D, ^2^D-NMR	[166]
10	Sisal; 650; 0.54 cotton linter; 410; 0.77	Ac_2_O	2:1; 110 °C; 4 h	1.5	5–7% LiCl/DMAC	SEC, ^1^H-NMR	[167]
11	Whatman CF-1 cellulose powder, 200	Cyclic lactones	4:1; 128 °C; 12 h	0.7	8% LiCl/DMAC	FT-Raman, ^13^C-NMR	[168]
12	Mercerized Cotton linter	Ac_2_O, NaOH	3:1; 110 °C; 1–5 h	1.1–2.2	6% LiCl/DMAC	SEM, ^1^H-NMR	[169]
13	MCC; 280	Carboxylic acids, CDI	3:1:3; 80 °C; 24–36 h	0.7	16.16% TBAF/DMSO; 5–10% LiCl/DMAC	^1^H-NMR	[140]
14	MCC; sisal	Ac_2_O	3:1; 110 °C; 1–4 h	1.6	5–9% LiCl/DMAC	^1^H-NMR, X-Ray	[170]
15	MCC; 260	Acyl-1*H*-benzotriazole	3:1; 60 °C; 3 h	1.07–1.89	11% TBAF/DMSO	FTIR, ^1^H-NMR	[144]
16	MCC; 100–200	Ac_2_O	6:1; 110 °C	1.5	6% LiCl/DMAC	SLS, ^1^H-NMR	[171]
17	MCC; cotton linter; sisal	Ac_2_O	0.5–6:1; 110 °C; 4 h	2.7	6% LiCl/DMAC	SLS, ^1^H-NMR	[69]
18	MCC	Acid anhydrides; diketene	1–3:1; 18 h; RT	1–2.8	8% LiCl/DMAC	X-Ray diffraction, ^13^C-NMR	[32]
19	MCC	Acid anhydrides	1–4.5:1; 18 h; 60 °C	1–2.8	8% LiCl/DMAC	Viscometry, X-Ray Diffraction	[33]
20	MCC; Cotton linters	Ac_2_O	0.5–12:1; 4 h, 110 °C	0.2–2.8	5–8% LiCl/DMAC	^13^C-NMR	[30]
21	MCC; 175; Sisal; 800	acid anhydride	4:1; 18 h; 60 °C	2.0	8% LiCl/DMAC	UV-Vis, FTIR	[172]
22	MCC; 175; eucalyptus; 1049	Acid anhydrides	6–13:1; 3 h; 60–100 °C	1.6–2.4	9% TBAF/DMSO	Viscometry, ^1^H-NMR	[142]
23	MCC; hard wood pulp	Butyric anhydride; diketene	3:1; 30–40 min; 110 °C	0.3–2.9	7% LiCl/DMAC	GPC, ^1^H,^13^C-NMR	[134]
24	MCC	Adipic anhydride	1–3:1; 2–20 h; 60–90 °C	2.1–2.6	5% LiCl/DMAC; 5% LiCl/DMI	^1^H-NMR, FTIR, SEC	[173]
25	Whatman CF-11; 190	Chloroacetic acid; TsCl	1:1; 24 h; 40–50 °C	1.5–2.6	9% LiCl/DMAC	^1^H, ^19^F-NMR	[174]
26	MCC; 300; Spruce sulfite pulp; 650	Acetic anhydride; vinyl carboxylates	2.3–10:1; 70 h; 40 °C	0.8–2.7	5% TBAF/DMSO	^1^H, ^13^C-NMR FTIR	[31]
27	Avicel; 260	Acid anhydrides, carboxylic acids, CDI	3:1; 3 h; 60 °C	1.18	7.5% LiCl/DMAC	FTIR, ^1^H-NMR	[175]
28	MCC; 280	α-lipoic acid, TsCl or CDI	3:1; 16 h 60 °C	1.45	LiCl/DMAC	FTIR, ^1^H-NMR	[176]
29	MCC; 150	Acid chlorides, DMAP	5–8:1; 3 h; 80 °C	2.8	6.7% LiCl/DMAC	FTIR, ^1^H-NMR; DSC, WAXS	[177]
30	Cellulose; 141	Ac_2_O	20:1; 2–24 h; 28–70 °C	1.65–2.85	1.6% LiCl/DMAC	Viscometry, FTIR	[178]
31	Cellulose	Acid chlorides	1:1; 8 h; 25 °C	2.4	9% LiCl/DMAC	Elemental analysis	[179]
32	Cellulose	Chloroacetyl chloride	6:1; 2 h; RT	2.8	10% LiCl/DMAC	FTIR, ^1^H-NMR	[180]
33	MCC Whatman CF-11	Carboxylic acids; TsCl	2: 24 h; 50 °C	2.8–2.9	4% LiCl/DMAC	^1^H-NMR, DSC	[127]
34	Cotton	Succinic anhydride, DMAP	20:1; 24 h; RT	2.5–2.6	8% LiCl/NMP; 15% TBAF/DMSO	Titration	[181]
35	MCC; 280	Acid chlorides	6:1; 24 h; RT	1.57	2.5% LiCl/DMAC	FTIR, WAXD, SEM	[182]
36	Whatman CF-11	Acid chloride, acetic anhydride pyridine	4 h; 60 °C	0.4–3.0	10% LiCl/DMAC	^13^C-NMR, UV-Vis	[183]
37	MCC; 280–5100	TsCl	0.6–9:1; 130 °C; 2 h	0.4–2.3	5% LiCl/DMAC	FTIR, ^13^C-NMR	[184]
***Nonionic and ionic ethers***							
38	MCC	*4,4'-*Bis(dimethylamino)-diphenylmethyl chloride	2:1; 50 °C; 24 h	0.54–1.05	DMAC, DMSO	UV, ^13^C-NMR	[185]
39	MCC; 280; Sulfite pulp; 680 Cotton linters; 1350	ClCH_2_CO_2_Na/NaOH	5:1; 70 °C; 48 h	2.07	1.7% LiCl/DMAC	HPLC, ^1^H, ^13^C-NMR	[186]
40	MCC; 330; Dissolving pulp; 950	ClCH_2_CO_2_Na/NaOH	10:5:1; 0.5–4 h, 70 °C	1.82–2.09	18.5% TBAF/DMSO; NMMNO/DMSO	^1^H, ^13^C-NMR, HPLC	[187]
41	Sisal; 574; Cotton linters; 400	Benzyl chloride, NaOH	3:1; 4 h; 70 °C	0.4–2.85	9% DMSO/TBAF	FTIR, ^1^H,^13^C-NMR, SEC	[151]
42	MCC	ClCH_2_CO_2_Na/NaOH	5:1; 48 h; 70 °C	2.07	LiCl/DMAC	FTIR, ^1^H-NMR	[61]
43	Sulfite pulp; 504	Allyl chloride, NaOH	36:1:36; 50 °C; 3 d	2.7	10.9% TBAF/ DMSO	^1^H-NMR, FTIR	[154]
44	MCC	ClCH_2_CO_2_Na/NaOH	2:1; 48 h; 70 °C	1.88	LiCl/DMAC	FTIR, ^1^H-NMR	[188]
45	Sisal; 640; 0.64; Linter; 400; 0.73	ClCH_2_CO_2_Na/NaOH	5:1:10; 70 °C; 4 h	2.17	9% TBAF/DMSO	SEC, HPLC, ^1^H-NMR	[189]
46	MCC	TDMSCl, Imidazole	4:1; 100 °C; 24 h	2.0	5% LiCl/DMAC	^1^H, ^13^C, COSY, HMQC, NMR	[190]
47	MCC; 419; Sulfite pulp; 560	TDMSCl, Imidazole	4:1:5; 100 °C; 24 h	1.98	8.4% LiCl/DMAC	^1^H-NMR, FTIR	[191]
48	MCC; 280	TDMSCl; allyl chloride; methyl iodide; benzyl chloride	RT; 42 h	1.92	LiCl/DMAC; TBAF/DMSO	^1^H,^13^C-NMR, UV-VIS, GPC	[192]
49	MCC	HMDS, TDMSCl	0.1:1:2; 80 °C; 1 h	2.89	LiCl/DMAC	FTIR, ^1^H-NMR	[193]
50	MCC; 117	TDMSCl, Imidazole	4.1:1; 24 h; 100 °C	2.06	7.8% LiCl/DMAC	FTIR, ^1^H-NMR	[194]
51	MCC	HMDS	8:1; 1 h; 80 °C	2.7–2.9	LiCl/DMAC	^1^H-NMR	[195]
***Miscellaneous***							
52	Avicel; 100–300	Lactones; N-carboxy α-amino acid anhydrides	2:1; 60 °C; 4 h		15% TBAF/DMSO	FTIR, GPC	[196]
53	Cotton linter; 640	Graphene	85 °C; 0.5 h		9% LiCl/DMAC	SEM, TGA	[197]
54	Cellulose	Phenyl isocyanate, pyridine	2.7:1; 12 h; RT	2.6	9% LiCl/DMAC	FTIR	[198]
55	MCC; 156; 290	ε-Caprolactam, *N*-methyl- ε-caprolactam, TsCl	5:1; 5 h; RT	0.12–1.17	9% LiCl/NMP	FTIR, ^1^H-NMR	[137]

**Table 4:** Dissolution and derivatization of chitin/chitosan and starch.

Entry	Polysaccharide	Dissolution conditions, temperature and time	Derivatizing agent	Reactions conditions Ratio derivatizing agent/AGU/cat.; T, °C; Reaction time, h	Yield; DS	Dissolution solvent	Study Techniques	Reference
**Chitin/chitosan**								
1	Chitin	RT, 1.5 h	----	------	-----	5% LiCl/DMAC	SEC, MALLS	[200]
2	Chitin	-----	------	------	-----	0.01–0.2% LiCl/DMAC	FTIR	[201]
3	Chitin, squid pens	RT, 120 h	-----	------	-----	5% LiCl/DMAC	Viscometry	[13]
4	Chitosan	------	------	6:1; 50 °C; 24 h	0.31	2.3% HCl/LiSCN	IR	[202]
5	Chitin, crab shell	RT, 5 d	Alkanoic acid, p-TsCl, Pyridine	8:1:8; 50 °C; 100 h	1.7–1.9	9% LiCl/DMAC	WAXD; FTIR, ^1^H-NMR	[203]
6	Chitin	RT, 3 h	Cyclic acid anhydrides	RT; 24 h	-----	5% LiCl/DMAC	Kinetic study	[204]
7	Chitin, crab shell	RT, 5 d	Acid chlorides, TEA	28:1; 50 °C; 6–48 h	0.97–1.77	7% LiCl/DMAC	GPC, ^1^H-NMR, FTIR	[205]
8	Chitin	------	Methacrylic acid, DCC, DMAP	6:1:6; RT; 48 h	-----	5% LiCl/DMAC	FTIR, NMR, SEM	[17]
9	Chitosan	150 °C, 0.5 h	ClCH_2_CO_2_Na/NaOH	----	----	9% LiCl/DMAC	FTIR, SEM, WAXS,	[206]
***Starch***								
10	Corn amylose	120 °C, 5 min	------	-------	-----	3% LiCl/DMAC	LS	[6]
11	Lign°Cellulose	-----	CrCl_2_	6:1; 100 °C; 5 h	----	10% LiCl/DMAC	HPLC, IEC	[1]
12	Potato starch	80 °C, 20 min	Acyl chlorides	3:1:3; 80 °C; 0.5 h	0.3–3	0.6% LiCl/DMAC	FTIR-Elemental analysis	[162]
13	Sago starch	-------	Succinic anhydride DMAP, Pyridine	3:1:3; 105 °C; 0.5–3 h	0.14–1.54	---------	FTIR, ^13^C-NMR	[163]
14	Potato starch	100 °C, 45 min	Fatty acid chlorides, CDI	1:1; 100 °C; 6 h	2.17	2% LiCl/DMAC	FTIR, ^1^H, ^13^C-NMR	[207]
15	Potato starch	120 °C, 2 h	Palmitoyl chloride, pyridine	4.5:1:5; 100 °C; 6 h	2.63	2% LiCl/DMAC	FTIR, ^1^H, ^13^C-NMR	[199]
16	Cassava starch	------	Maleopimaric acid chloride Maleopimaric acid, pyridine	115 °C; 2 h	0.11–0.17	DMF/pyridine; Microwave	^1^H, ^13^C-NMR, FTIR	[208]
17	Maize starch	80 °C, 2 h	TDMSCl, Pyridine	0.6–6:1; 20 °C; 20 h	1.8	DMSO	^1^H-NMR	[209]
18	Potato starch	−20 °C, 1–2 h, 20 °C, 24 h,	TDMSCl/NMP	1.6–4:1; 20 °C; 24 h	1.0	12–19% NMP/ammonia	^1^H-NMR	[210]
19	Amylose, amylopectin	-----	1. NaN_3_, 2. PPh_3_, CBr_4_	10:1; 100 °C; 1 h	------	8% LiCl, LiBr/DMF	IR, ^1^H, ^13^C-NMR	[14]

## Abbreviations and Symbols

AGU: anhydroglucose unit; AdCl: adamantoyl chloride; CA: cellulose acetate; CDI: *N,N*-carbonyldiimidazole; Cell: cellulose; CMC: carboxymethyl cellulose; DAS: dipolar aprotic solvent; DCC: dicyclohexyl carbodiimide; DMAC: *N,N*-dimethylacetamide; DMAP: 4-(*N,N*-dimethylamino)pyridine; DMI:1,3-dimethyl-2-imidazolidinone; DMSO: dimethyl sulfoxide; DLS: dynamic- or quasi-elastic light scattering; DS: degree of substitution of the polysaccharide derivative; ESI-TS: dlectrospary ionization, thermospray; EWNN: alkaline solution of iron sodium tartrate; FAB: fast-atom bombardment; GPC: gel permeation chromatography; HMDS: 1,1,1,3,3,3-hexamethyldisilazane; HRS: homogeneous reaction scheme; Ic: index of crystallinity of the biopolymer; IL: ionic liquid; LS: light scattering; MALLS: multiple-angle laser light scattering; MAPMCl: *4,4'*-bis(dimethylamino)diphenylmethyl chloride; MCC: microcrystalline cellulose; NMP: *N*-methyl-2-pyrrolidinone; RT: room temperature; SAXS: small-angle X-ray scattering; SE: strong electrolyte; SEM: scanning electron microscopy; SLS: static light scattering; TBAF: tetra(1-butyl)ammonium fluoride trihydrate; TDMSCl: thexyldimethylchlorosilane; TEA: triethylamine; TEM: transmission electron microscopy; TsCl: tosyl chloride; WAXD: wide-angle X-ray diffraction.
